# Supplementary material for: Measuring the Residual Levels of Fenpyroximate and Its Z-Isomer in Citrus Using Ultra-High-Performance Liquid Chromatography–Tandem Mass Spectrometry and Assessing the Related Dietary Intake Risks
Source: Molecules. 2023 Oct 17;28(20):7123. doi: 10.3390/molecules28207123 (PMC10609617; doi:10.3390/molecules28207123)
Supplement: Supplementary file 1 [file molecules-28-07123-s001.zip › molecules-2634264-supplementary.pdf]

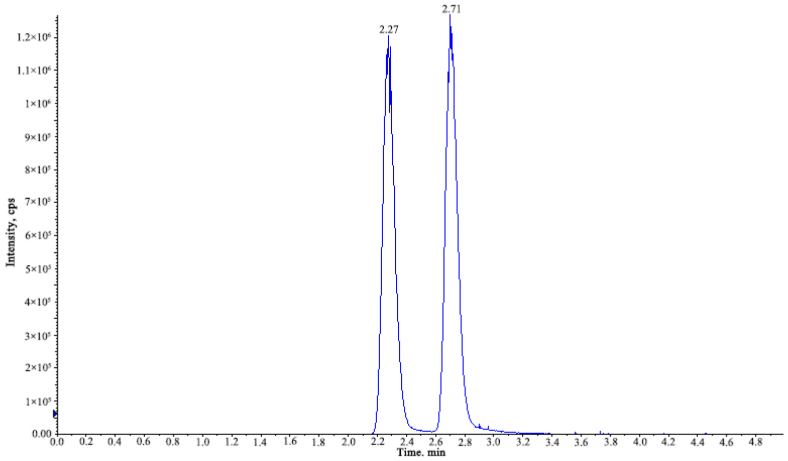

Figure S1. Total ion chromatogram of fenpyroximate and Z-fenpyroximate.

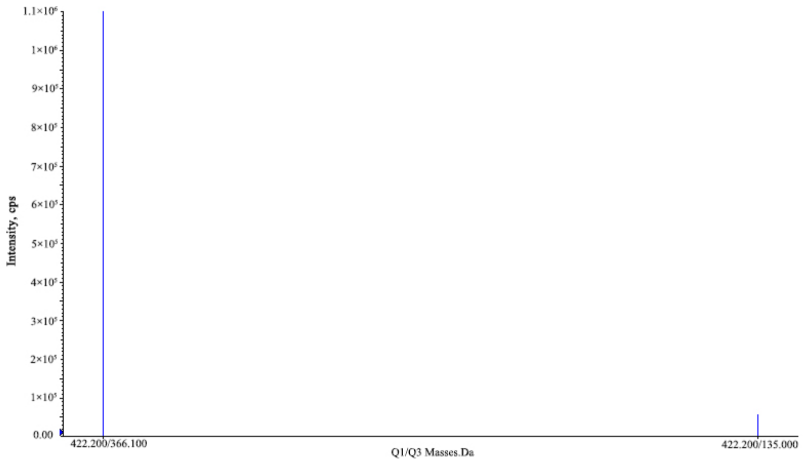

Figure S2. MS spectra of fenpyroximate and Z-fenpyroximate.

Table S1. Soil properties and climatic conditions of field experiment

Location	Soil properties			Climatic conditions	
	PH	Organic matter content(%)	Cation exchange capacity(cmol/kg)	Average temperature(℃)	Total precipitation (mm)
Gansu	7.00	2.55	3.90	16.6	57.0
Zhejiang	7.51	2.31	13.60	15.0	37.1
Zhangjiajie in Hunan	5.15	2.64	20.40	18.2	25.7
Changsha in Hunan	5.90	2.50	10.24	26.5	20.8
Hubei	7.82	1.23	12.00	7.2	0.0
Guangxi	4.50	3.79	4.70	13.4	7.9
Chongqing	6.50	2.80	16.30	20.0	0.0
Guizhou	5.10	9.22	22.17	11.8	20.9
Yunnan	5.00	2.26	16.90	12.9	25.0
Guangzhou in Guangdong	5.68	1.83	14.91	16.8	0.0
Maoming in Guangdong	6.11	1.87	11.67	20.0	0.0
Hainan	4.72	2.21	8.25	22.2	60.9

Table S2. Quality control (QC) during actual sample test

Matrix	Compounds	Detected date	Spiked Level (mg/kg)	Average recovery (n = 2)/%	RSD(%)
Whole citrus	Fenpyroximate	2023.6.17	0.1	113	2
	Z-fenpyroximate			99	1
Peeled citrus	Fenpyroximate	2023.6.19	0.1	108	3
	Z-fenpyroximate			97	3

Table S3. Final residues of fenpyroximate, Z-fenpyroximate, and total residues in citrus.

Field	Pre -Harvest Interval (Days)	Residues in whole citrus(mg kg^{-1})			Residues in flesh citrus(mg kg^{-1})		
		Fenpyroximate	Z-fenpyroximate	Total Residues (Evaluate Definition)	Fenpyroximate	Z-fenpyroximate	Total Residues (Evaluate Definition)
Gansu	15	<0.010, <0.010	<0.010, <0.010	<0.020, <0.020	<0.010, <0.010	<0.010,<0.010	<0.020,<0.020
	25	<0.010, <0.010	<0.010, <0.010	<0.020, <0.020	<0.010, <0.010	<0.010,<0.010	<0.020,<0.020
Zhejiang	15	0.15, 0.18	<0.010, <0.010	0.16, 0.19	<0.010, <0.010	<0.010,<0.010	<0.020,<0.020
	25	0.11, 0.054	<0.010, <0.010	0.12, 0.064	<0.010, <0.010	<0.010,<0.010	<0.020,<0.020
Zhangjiajie	15	0.15, 0.15	<0.010, <0.010	0.16, 0.16	<0.010, <0.010	<0.010,<0.010	<0.020,<0.020
	25	0.12, 0.12	<0.010, <0.010	0.13, 0.13	<0.010, <0.010	<0.010,<0.010	<0.020,<0.020
Changsha	15	0.034,0.035	<0.010, <0.010	0.044, 0.045	0.012, 0.012	<0.010,<0.010	0.022, 0.022
	25	0.032, 0.018	<0.010, <0.010	0.042, 0.028	0.018, 0.019	<0.010,<0.010	0.028, 0.029
Hubei	15	0.13, 0.13	<0.010, <0.010	0.14, 0.14	0.047, 0.045	<0.010,<0.010	0.057, 0.055
	25	0.11, 0.10	<0.010, <0.010	0.12, 0.11	0.040, 0.030	<0.010,<0.010	0.050, 0.040
Guangxi	15	0.039, 0.043	<0.010, <0.010	0.049, 0.053	<0.010, <0.010	<0.010,<0.010	<0.020,<0.020
	25	0.050, 0.056	<0.010, <0.010	0.060, 0.066	<0.010, <0.010	<0.010,<0.010	<0.020,<0.020
Chongqing	15	0.18, 0.13	<0.010, <0.010	0.19, 0.14	<0.010, <0.010	<0.010,<0.010	<0.020,<0.020
	25	0.072, 0.14	<0.010, <0.010	0.082, 0.15	<0.010, <0.010	<0.010,<0.010	<0.020,<0.020
Guizhou	15	0.17, 0.094	<0.010, <0.010	0.18, 0.10	0.063, 0.063	<0.010,<0.010	0.073, 0.073
	25	0.17, 0.094	<0.010, <0.010	0.18, 0.10	0.041, 0.030	<0.010,<0.010	0.051, 0.040
Yunnan	15	0.085, 0.097	<0.010, <0.010	0.095, 0.11	0.021, 0.022	<0.010,<0.010	0.031, 0.032
	25	0.14, 0.13	<0.010, <0.010	0.15, 0.14	<0.010, <0.010	<0.010,<0.010	<0.020,<0.020
Guangzhou	15	0.015, 0.016	<0.010, <0.010	0.025, 0.026	<0.010, <0.010	<0.010,<0.010	<0.020,<0.020
	25	<0.010, <0.010	<0.010, <0.010	<0.020, <0.020	<0.010, <0.010	<0.010,<0.010	<0.020,<0.020

Maoming	15	0.024, 0.029	<0.010, <0.010	0.034, 0.039	<0.010, <0.010	<0.010,<0.010	<0.020,<0.020
	25	0.026, 0.032	<0.010, <0.010	0.036, 0.042	<0.010, <0.010	<0.010,<0.010	<0.020,<0.020
Hainan	15	0.062, 0.084	<0.010, <0.010	0.072, 0.094	<0.010, <0.010	<0.010,<0.010	<0.020,<0.020
	25	0.080, 0.028	<0.010, <0.010	0.090, 0.038	0.011, 0.012	<0.010,<0.010	0.021, 0.022
